# Jottings

**Published:** 1910-01-22

**Authors:** 


### Jottings.

Miss Sarah Jones died last week in the Royal Hospital for Incurables, Putney Heath, after being an in-patient of that institution for 52 years.

The board of management of the Anti-Vivisection Battersea General Hospital on January 11 appointed a house surgeon and a physician to the institution. A medical officer, therefore, will be always in attendance.

Ear, Nose, and Throat Hospital.—The Duke of Devonshire has accepted the office of patron of the Metropolitan Ear, Nose, and Throat Hospital, Grafton Street, Fitzroy Square, rendered vacant by the death of Lord Ripon.

Princess Christian has consented to open the bazaar in aid of the Rebuilding Fund of the Hospital for Women, Soho Square, W., in May. The hospital is now rebuilding, at a cost of £20,000, and about £12,500 has already been received.

The new building in Richmond Row as the East branch of the Liverpool Dispensaries was opened by Lord Derby on January 13. The old building could not meet the recent demands upon it, represented last year by 40,000 attendances, or 17,000 patients.

The Hospital for Sick Children, Toronto, Canada, has just received the splendid sum of $10,000 (£2,000) as a Christmas present from Mr. John Ross Robertson, the publisher, whose purse and newspapers have been placed for twenty years now at the service of this institution.

At a meeting of managers of the Metropolitan Asylum District held last Saturday, a letter was received from the Southwark Guardians agreeing with the proposal that the managers should use vacant isolation hospitals for consumptive cases needing sanatorium treatment. The board accepted the recommendation of the Children's Committee to add to its seaside homes for children by purchasing property adjacent to the existing institution, East Cliff House, Margate, for £2,350. From the taking over of East Cliff House by the board in 1897 2,833 children have been to the home, and the most satisfactory reports as to the curative results received.

"I notice under wills in to-day's (January 8) edition of the *Times* that someone has revoked a gift left to a most excellent charity, giving as his reason 'as I do not feel that they are justified in spending thousands a year beyond their income.'" . . . "There seems to be a growing tendency to give to charities which exceed their income to the exclusion of those who refuse to live beyond their means. As a treasurer of one of our smaller charities I have often been told if only I would agree to give away a little more than our yearly income that we should be much more generously supported by the public; which I hope is not the case, but am rather afraid that it may be true."—Mr. Douglas Hankey, in the *Times* of January 12.

The Hospital Saturday Fund for the past year closed on Monday. The receipts from January 12, 1909, to the 10th inst. inclusive amounted to £30,383, an increase of £553 on last year. Since its foundation in 1873, the total contributions of the metropolis have reached £603,493. The main portion has been subscribed by the industrial classes.

An analysis has been made in the *National Church* of the share which each religious organisation has had in making up the £39,118 3s. 4d. which was collected on Hospital Sunday in London last year. Apart from the Church of England, which naturally heads the list, the other bodies reached four or three figures in the following order: Congregationalists, Jews, Wesleyans, Presbyterians, Baptists, Roman Catholics, Unitarians, Church of Scotland, and the Theistic Church.

